# Supplementary material for: Operational resilience: concepts, design and analysis
Source: Sci Rep. 2016 Jan 19;6:19540. doi: 10.1038/srep19540 (PMC4726063; doi:10.1038/srep19540)
Supplement: Supplementary Information [file srep19540-s1.pdf]

# Operational resilience: concepts, design and analysis

**Alexander A. Ganin[1,2], Emanuele Massaro[1,3,+], Alexander Gutfraind[4], Nicolas Steen[1], Jeffrey M. Keisler[5], Alexander Kott[6], Rami Mangoubi[7], and Igor Linkov[1,*]**

[1]U.S. Army Corps of Engineers – Engineer Research and Development Center, Environmental Laboratory, Concord, MA, 01742, USA
[2]University of Virginia, Department of Systems and Information Engineering, Charlottesville, VA, 22904, USA
[3]Carnegie Mellon University, Department of Civil and Environmental Engineering, Pittsburgh, PA, 15213, USA
[4]University of Illinois at Chicago, School of Public Health, Chicago, IL, 60612, USA
[5]University of Massachusetts Boston, College of Management, Boston, MA, 02125, USA
[6]U.S. Army Research Laboratory, Network Science Division, Adelphi, MD, 20783, USA
[7]Charles Stark Draper Laboratory, Cambridge, MA, 02139, USA
[*]Igor.Linkov@usace.army.mil
[+]presently at Massachusetts Institute of Technology, Senseable City Laboratory, Cambridge, MA, 02139, USA

## S1. Survey of Existing Approaches to Resilience Quantification

In this section, we focus on definitions and applications of resilience, especially as it pertains to disaster management and engineering. For example, Bruneau et al.[1] identified four dimensions of seismic community resilience: technical, organizational, social, and economic. They integrated different measures of resilience – robustness, rapidity, resourcefulness, and redundancy – in order to minimize the probability of system failures, the consequences arising from such failures, and the time it takes for recovery. The definition proposed by the US Department of Defense[2,3] is useful, except that it presupposes that the metrics are independent of one another and does not contain an explicit consideration of time (but can be extended to do so).

The resilience of complex systems is often quantified as the probability of failure under a specific threat scenario at a specified time. This approach basically adds a temporal dimension to traditional risk assessment. For example, Cimellaro et al.[4] defined resilience quantitatively as the normalized area underneath a function $Q(t)$ that expresses the system's critical functionality as a function of time. Furthermore, they enhanced the definition of the resilience properties proposed by Bruneau et al.[1] by introducing the quantitative measures of control time, $T_{LC}$, and recovery time, $T_{RE}$.

$$R = \int_{t_e}^{t_e+T_{LC}} \frac{Q(t)}{T_{LC}} dt \tag{S1.1}$$

Utilizing nonlinear loss functions and recovery functions, the factors of $Q(t)$ are quantified, and resilience is derived as a dimensionless measure that expresses the system's functionality over time. The use of fragility curves for the loss estimation models is another feature of the proposed quantification.

Ouyang et al.[5] proposed an expected, time-dependent, annual resilience metric that measures the system's preparedness and capacity to confront and recover from the occurrence of different types of hazards. The aforementioned system properties are collectively regarded as the system's performance; therefore, the metric provides a performance curve that plots a time-dependent graph, the area of which represents the system's resilience. The metric is conceptually similar to other proposals, since it is based on stochastic modeling of a "hazard occurrence – restoration actions – recovery" iterative process; however, it differs from other proposals in that it introduces the notion of quantifying a system's resilience under multiple hazards.

Bocchini and Frangopol[6] devise a model of recovery planning for networks of highway bridges that have been damaged due to an earthquake. In their model, optimal bridge restoration activities are identified so as to maximize resilience, minimize the time required to return the network to a targeted functionality level, and minimize the cost of these activities. The equation they employ to measure resilience under these constraints is as follows:

$$Q_2 = \frac{\int_{t_1}^{t_2} F(t) dt}{t_2 - t_1} \tag{S1.2}$$

Note that $F$ represents the system performance measure. The time at which the system is disrupted is $t_1$, and the time at which the system achieves a targeted level of performance is $t_2$, at which time recovery is considered complete. This approach is similar to Bruneau's resilience loss calculation with one noteworthy difference: Bocchini and Frangopol measure the area between $F(t)$ and 0 – as opposed to measuring the area between $F_0$ and $F(t)$ – and then normalize over the recovery time period. This difference is important because it increases the value of resilience by simply increasing the value of $Q_2$. Bruneau's approach has the opposite outcome. We generalize and expand the realm of this definition. For instance, for networks, we incorporate such factors as the relative importance of nodes, links, and the weights $w_i(t)$; and the level of damage done to a component, or $\pi_i(t)$.

Vugrin et al.[7] proposed a resilience framework that depends upon the calculation of two key quantities: the systemic impact ($SI$) – that is, the cumulative impact of decreased system performance after a disruptive event – and the total recovery effort ($TRE$) – that is, the cumulative resources expended in recovery activities. These authors utilize these quantities to devise a composite resilience metric:

$$Z = SI + \alpha TRE \tag{S1.3}$$

Note that $\alpha$ is a weighting factor whose function is to take into accounts both unit conversion and relative weighting between $SI$ and $TRE$ in overall evaluation. This framework can be applied to transportation networks, in addition to continuous, dynamic systems and agent-based models. We also attempt to generalize this definition. In network science, focus is often on multilayer networks[8–10] on which it is intuitive to emulate socio-technical systems, such as human-information networks,[11,12] and to comprehend the interactions among interdependent infrastructures.[13–17] In many of these studies, the focus is on robustness or the percolation process during or after the occurrence of an adverse event,[18] which constitutes the first phase of our resilience definition, that is, when the critical functionality in Figure 1 is decreasing. Recently, some authors have been focusing on the recovery phase and on self-healing processes in complex networks,[19,20] which constitutes the second or recovery phase of our definition of resilience, that is, when the critical functionality is increasing. Our definition, therefore, embodies both the concept of robustness and the concept of recovery.

In Ref. 21 a different metric for quantifying the resilience or efficiency of interconnected networks is outlined. The authors introduce a mechanism to perform such exploration, using random walks on multilayer networks, and they show how the topological structure, together with the navigation strategy, influences the efficiency of exploring the whole structure. They quantify the efficiency of the system as the number of sites visited by a random walker during a certain temporal window. They define the coverage $\rho(t)$ as the average fraction of distinct vertices visited at least once in a period of time less than or equal to $t$. Roughly speaking, the resilience of an interconnected system is a function of the fraction $\phi$ of random failures. In particular, they define the resilience $r(\phi)$ as:

$$r(\phi) = \frac{\langle \rho_\phi(\tau) \rangle}{\rho_0(\tau)} \tag{S1.4}$$

where $\rho_\phi(\tau)$ is the coverage at time $t$ of the network subject to $\phi$ failures. The averages are calculated over several random realizations of the failures.

However, another area of research is more heavily concentrated on defining all of the temporal aspects of resilience without consideration of the complex interactions among the different layers. Scholars in engineering resilience and, in particular, transportation systems are presently examining whether graph theory is a viable way to quantify resilience.[22–26]

Table S1.1 displays which element(s) of the resilience matrix are contained in the complex network-related literature, as related to criteria advanced in two sources: the National Academy of Science (NAS), and the Network Centric Warfare (NCW) domains.

| References | NAS Phases | | | | NCW Domains | | | |
|---|---|---|---|---|---|---|---|---|
| | Plan | Absorb | Recover | Adapt | Physical | Information | Cognitive | Social |
| 1,4,6,7 | | x | x | | x | | | |
| 5 | x | x | x | | x | | | |
| 13–21,27 | | x | | | x | | | |
| 8–10,28,29 | | x | | | x | | | x |

**Table S1.1.** Networked System Approaches and Their Disposition on the Resilience Matrix.

## References


1. Bruneau, M. *et al.* A framework to quantitatively assess and enhance the seismic resilience of communities. *Earthquake Spectra* **19**, 733–752, doi:10.1193/1.1623497 (2003).
2. Alberts, D. S. *Understanding Information Age Warfare* (CCRP Publication Series, 2001).
3. Smith, E. A. *Complexity, Networking, and Effects-Based Approaches to Operations* (Center for Advanced Concepts and Technology, 2006).
4. Cimellaro, G. P., Reinhorn, A. M. & Bruneau, M. Framework for analytical quantification of disaster resilience. *Engineering Structures* **32**, 3639–3649, doi:10.1016/j.engstruct.2010.08.008 (2010).
5. Ouyang, M., Dueñas-Osorio, L. & Min, X. A three-stage resilience analysis framework for urban infrastructure systems. *Structural Safety* **36-37**, 23–31, doi:10.1016/j.strusafe.2011.12.004 (2012).
6. Bocchini, P. & Frangopol, D. M. Optimal resilience- and cost-based postdisaster intervention prioritization for bridges along a highway segment. *Journal of Bridge Engineering* **17**, 117–129, doi:10.1061/(ASCE)BE.1943-5592.0000201 (2012).
7. Vugrin, E. D., Warren, D. E., Ehlen, M. A. & Camphouse, C. R. A framework for assessing the resilience of infrastructure and economic systems in *Sustainable and Resilient Critical Infrastructure Systems Simulation, Modeling, and Intelligent Engineering* (eds. Gopalakrishnan, K. & Peeta, S.) 77–116 (Springer, 2010).
8. Bianconi, G. Multilayer networks: dangerous liaisons? *Nature Physics* **10**, 712–714, doi:10.1038/nphys3097 (2014).
9. De Domenico, M. *et al.* Mathematical formulation of multilayer networks. *Physical Review X* **3**, doi:10.1103/PhysRevX.3.041022 (2013).
10. Kivela, M. *et al.* Multilayer networks. *Journal of Complex Networks* **2**, 203–271, doi:10.1093/comnet/cnu016 (2014).
11. Bagnoli, F. & Massaro, E. Risk perception and epidemic spreading in multiplex networks in *ISCS 2014: Interdisciplinary Symposium on Complex Systems*, Vol. 14 (eds. Sanayei, A., E. Rössler, O. & Zelinka, I.) 319–332 (Springer International Publishing, 2015).
12. Granell, C., Gómez, S. & Arenas, A. Dynamical interplay between awareness and epidemic spreading in multiplex networks. *Physical Review Letters* **111**, doi:10.1103/PhysRevLett.111.128701 (2013).
13. Buldyrev, S. V., Parshani, R., Paul, G., Stanley, H. E. & Havlin, S. Catastrophic cascade of failures in interdependent networks. *Nature* **464**, 1025–1028, doi:10.1038/nature08932 (2010).
14. Gao, J., Buldyrev, S. V., Stanley, H. E. & Havlin, S. Networks formed from interdependent networks. *Nature Physics* **8**, 40–48, doi:10.1038/nphys2180 (2011).
15. Brummitt, C. D., D'Souza, R. M. & Leicht, E. A. Suppressing cascades of load in interdependent networks. *Proceedings of the National Academy of Sciences* **109**, E680–E689, doi:10.1073/pnas.1110586109 (2012).
16. Parshani, R., Buldyrev, S. V. & Havlin, S. Interdependent networks: reducing the coupling strength leads to a change from a first to second order percolation transition. *Physical Review Letters* **105**, doi:10.1103/PhysRevLett.105.048701 (2010).
17. Gao, J., Buldyrev, S. V., Havlin, S. & Stanley, H. E. Robustness of a network of networks. *Physical Review Letters* **107**, doi:10.1103/PhysRevLett.107.195701 (2011).
18. Callaway, D. S., Newman, M. E. J., Strogatz, S. H. & Watts, D. J. Network robustness and fragility: percolation on random graphs. *Physical Review Letters* **85**, 5468–5471, doi:10.1103/PhysRevLett.85.5468 (2000).
19. Majdandzic, A. *et al.* Spontaneous recovery in dynamical networks. *Nature Physics* **10**, 34–38, doi:10.1038/nphys2819 (2013).
20. Quattrociocchi, W., Caldarelli, G. & Scala, A. Self-healing networks: redundancy and structure. *PLoS ONE* **9**, doi:10.1371/journal.pone.0087986 (2014).
21. De Domenico, M., Sole-Ribalta, A., Gomez, S. & Arenas, A. Navigability of interconnected networks under random failures. *Proceedings of the National Academy of Sciences* **111**, 8351–8356, doi:10.1073/pnas.1318469111 (2014).
22. Baños, R., Reca, J., Martínez, J., Gil, C. & Márquez, A. L. Resilience indexes for water distribution network design: a performance analysis under demand uncertainty. *Water Resources Management* **25**, 2351–2366, doi:10.1007/s11269-011-9812-3 (2011).
23. Baroud, H., Barker, K., Ramirez-Marquez, J. E. & Rocco S., C. M. Importance measures for inland waterway network resilience. *Transportation Research Part E: Logistics and Transportation Review* **62**, 55–67, doi:10.1016/j.tre.2013.11.010 (2014).

**24.** Tcholtchev, N., Grajzer, M. & Vidalenc, B. Towards a unified architecture for resilience, survivability and autonomic fault-management for self-managing networks in *Service-Oriented Computing. ICSOC/ServiceWave 2009 Workshops*, Vol. 6275 (eds. Hutchison, D. *et al.*) 335–344 (Springer Berlin Heidelberg, 2010).

**25.** Como, G., Savla, K., Acemoglu, D., Dahleh, M. A. & Frazzoli, E. Stability analysis of transportation networks with multiscale driver decisions in *Proceedings of American Control Conference (ACC)* 2436–2441 (IEEE, 2011).

**26.** Smith, P. *et al.* Network resilience: a systematic approach. *IEEE Communications Magazine* **49**, 88–97, doi:10.1109/MCOM.2011.5936160 (2011).

**27.** Van Mieghem, P. *et al*, *A Framework for Computing Topological Network Robustness*. *Technical Report*. (2010) Available at: http://www.nas.ewi.tudelft.nl/images/stories/javier/report20101218_A_Framework_for_Computing_Topological_Robustness.pdf. (Accessed: 9th April 2015)

**28.** Massaro, E. & Bagnoli, F. Epidemic spreading and risk perception in multiplex networks: A self-organized percolation method. *Physical Review E* **90**, doi:10.1103/PhysRevE.90.052817 (2014).

**29.** Granell, C., Gómez, S. & Arenas, A. Competing spreading processes on multiplex networks: Awareness and epidemics. *Physical Review E* **90**, doi:10.1103/PhysRevE.90.012808 (2014).

## S2. Dependency of the Resilience on the Redundancy and Switching Probabilities for Selected Classes of Attacks

In this section, we provide additional results that reinforce the observations in the Results and Conclusion section. Specifically, with a fixed recovery time, the redundancy and switching probabilities pm and $p_s$, respectively, can be traded off to maintain a desired level of resilience. Moreover, increasing both parameters has an additive effect on resilience. Figures S2.1 – S2.4 illustrate this point.

In Figure S2.5, the switching probability is held constant, and we see that $T_R$ and $p_m$ can also be traded off to maintain a desired resilience level. The additive effect of this parameter pair is also evident.

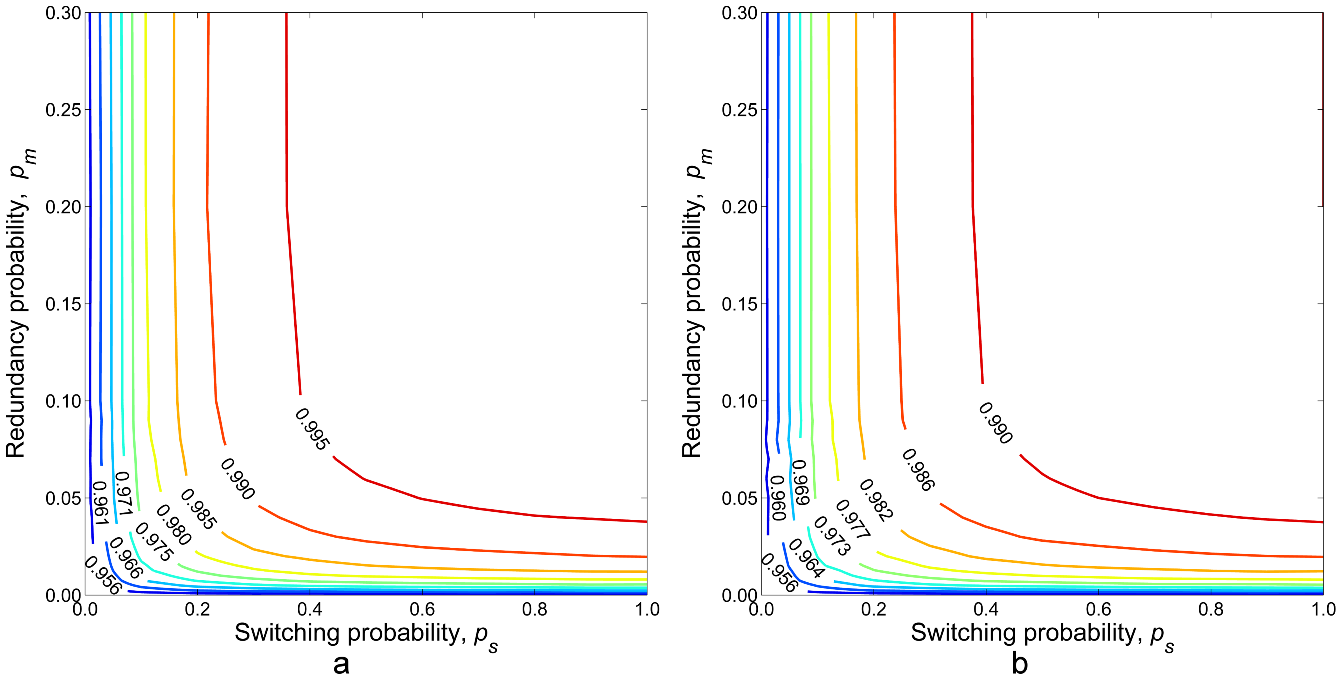


**Figure S2.1.** *Resilience as a function of design parameters.* Resilience (value shown on curves) dependencies on switching probability at each time step, or $p_s$, and redundancy parameter $p_m$, for a four level hierarchical network where the initial number of destroyed nodes at each level is $I_0^0 = 1$, $I_1^0 = I_2^0 = I_3^0 = 0$ respectively, and recovery time is held constant at $T_R = 0.5T_C$ with instant ($\sigma \in [1.1\text{E-}5; 1.7\text{E-}2]$) switching (a) and one-step delayed ($\sigma \in [1.6\text{E-}3; 1.7\text{E-}2]$) switching (b).

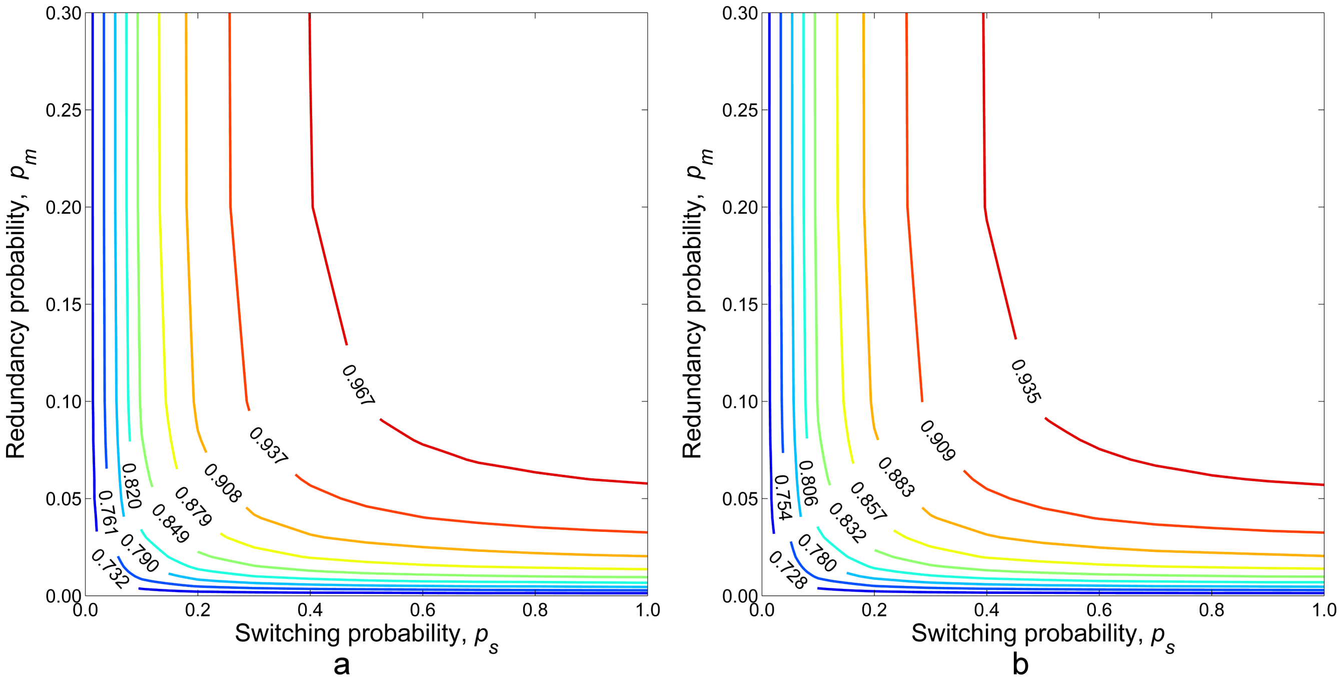


**Figure S2.2.** *Resilience as a function of design parameters.* Resilience (value shown on curves) dependencies on switching probability at each time step, or $p_s$, and redundancy parameter $p_m$, for a four level hierarchical network where the initial number of destroyed nodes at each level is $I_0^0 = 8$, $I_1^0 = I_2^0 = I_3^0 = 0$ respectively, and recovery time is held constant at $T_R = 0.5T_C$ with instant ($\sigma \in [1.0\text{E-}4; 2.5\text{E-}2]$) switching (a) and one-step delayed ($\sigma \in [3.3\text{E-}3; 2.4\text{E-}2]$) switching (b).

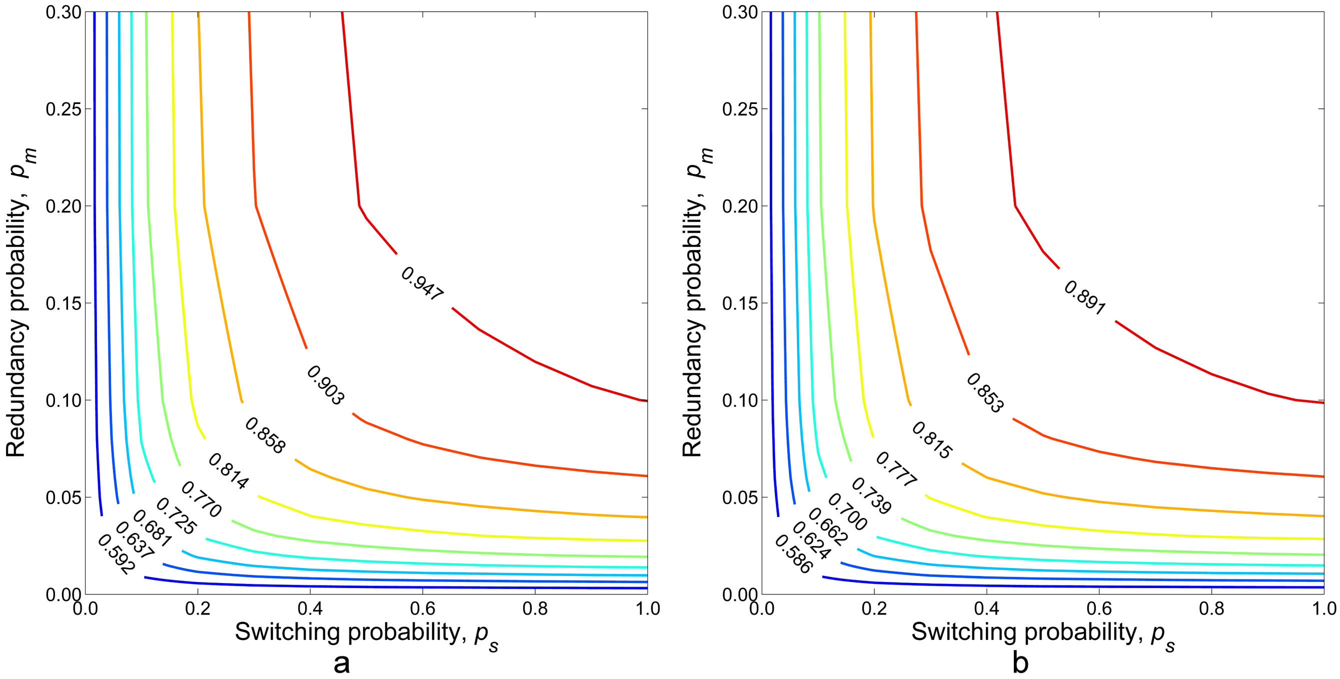


**Figure S2.3.** *Resilience as a function of design parameters.* Resilience (value shown on curves) dependencies on switching probability at each time step, or $p_s$, and redundancy parameter $p_m$, for a four level hierarchical network where the initial number of destroyed nodes at each level is $I_0^0 = 16$, $I_1^0 = I_2^0 = I_3^0 = 0$ respectively, and recovery time is held constant at $T_R = 0.5T_C$ with instant ($\sigma \in [6.5\text{E-}4; 2.0\text{E-}2]$) switching (a) and one-step delayed ($\sigma \in [3.1\text{E-}3; 2.0\text{E-}2]$) switching (b).

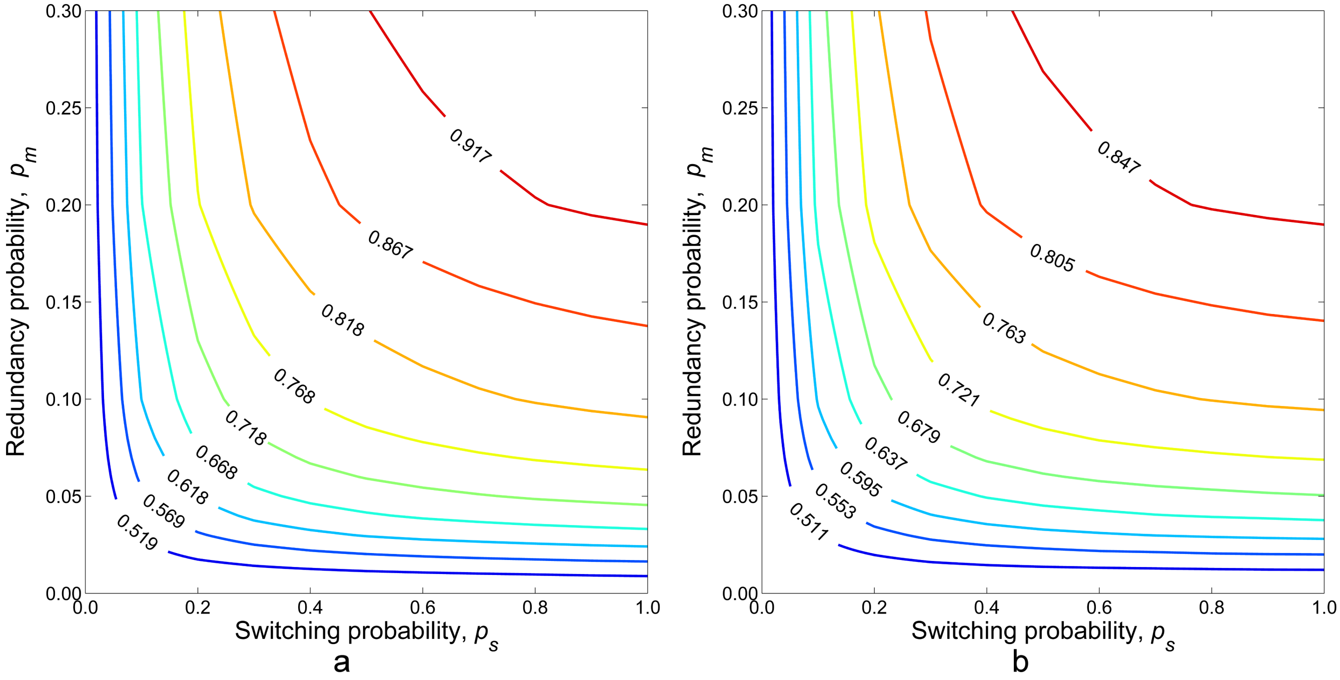


**Figure S2.4.** *Resilience as a function of design parameters.* Resilience (value shown on curves) dependencies on switching probability at each time step, or $p_s$, and redundancy parameter $p_m$, for a four level hierarchical network where the initial number of destroyed nodes at each level is $I_0^0 = 24$, $I_1^0 = I_2^0 = I_3^0 = 0$ respectively, and recovery time is held constant at $T_R = 0.5T_C$ with instant ($\sigma \in [3.2\text{E-}3; 1.7\text{E-}2]$) switching (a) and one-step delayed ($\sigma \in [3.3\text{E-}3; 1.6\text{E-}2]$) switching (b).

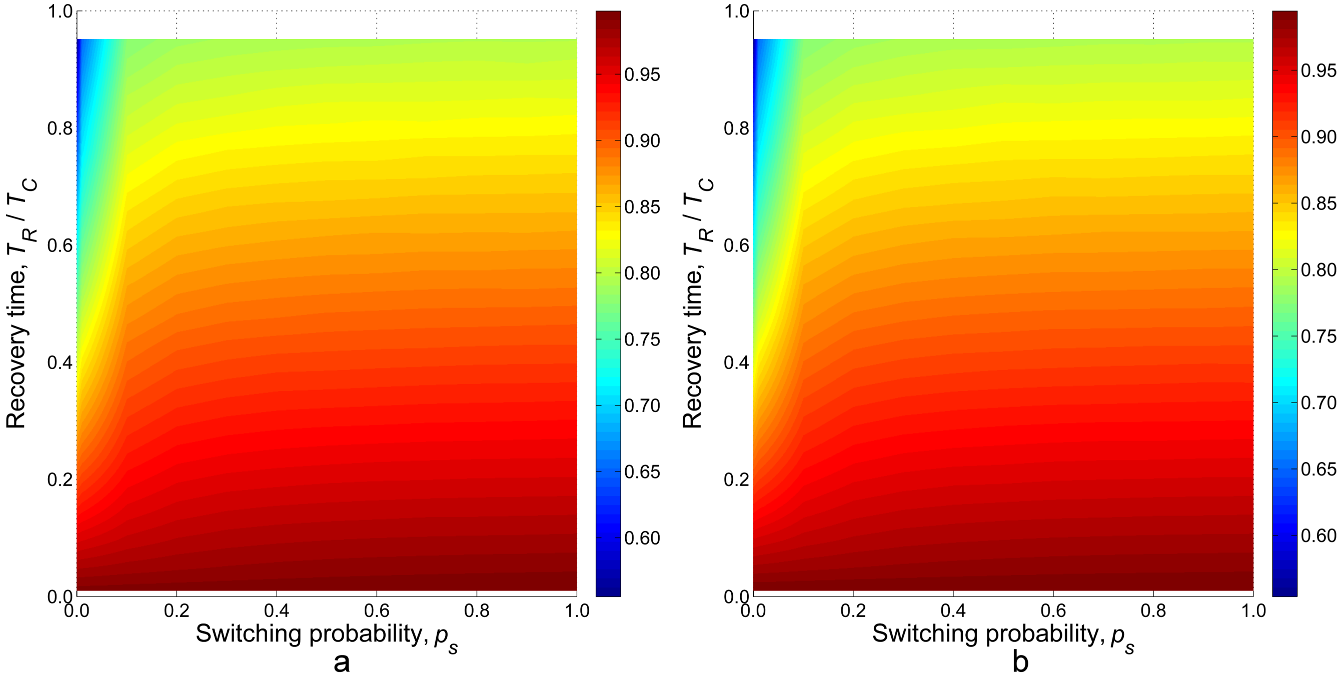


**Figure S2.5.** *Resilience as a function of design parameters.* Resilience (color bar indicates the value) dependencies on switching probability at each time step, or $p_s$, and recovery time $T_R$ for a four level hierarchical network where the initial number of destroyed nodes at each level is $I_0^0 = I_1^0 = I_2^0 = I_3^0 = 5$ respectively and redundancy parameter $p_m = 0.01$ with instant ($\sigma \in [2.4\text{E-}4; 4.2\text{E-}2]$) switching (a) and one-step delayed ($\sigma \in [5.9\text{E-}4; 4.3\text{E-}2]$) switching (b).

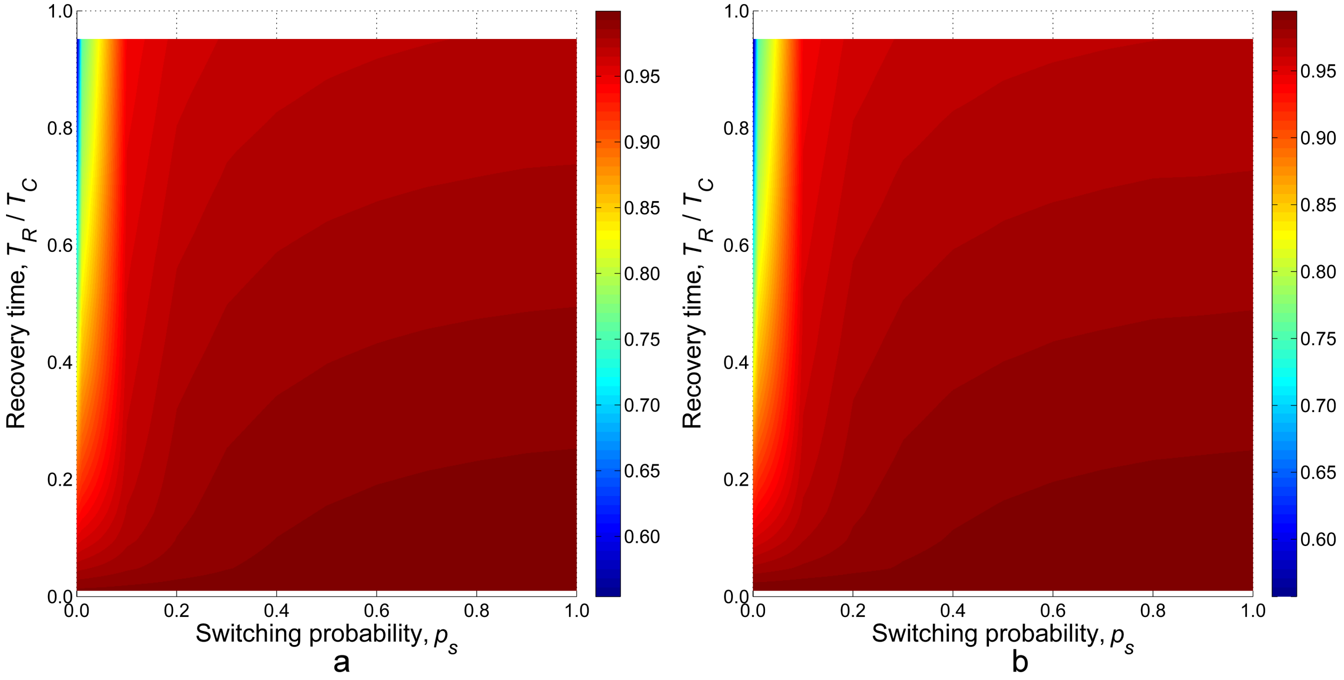


**Figure S2.6.** *Resilience as a function of design parameters.* Resilience (color bar indicates the value) dependencies on switching probability at each time step, or $p_s$, and recovery time $T_R$ for a four level hierarchical network where the initial number of destroyed nodes at each level is $I_0^0 = I_1^0 = I_2^0 = I_3^0 = 5$ respectively and redundancy parameter $p_m = 0.1$ with instant ($\sigma \in [3.2\text{E-}5; 4.3\text{E-}2]$) switching (a) and one-step delayed ($\sigma \in [5.6\text{E-}4; 4.3\text{E-}2]$) switching (b).
